# MetaCHIP: community-level horizontal gene transfer identification through the combination of best-match and phylogenetic approaches

**DOI:** 10.1186/s40168-019-0649-y

**Published:** 2019-03-04

**Authors:** Weizhi Song, Bernd Wemheuer, Shan Zhang, Kerrin Steensen, Torsten Thomas

**Affiliations:** 1 Centre for Marine Bio-Innovation, University of New South Wales, Sydney, NSW 2052 Australia; 2 School of Biotechnology and Biomolecular Sciences, University of New South Wales, Sydney, NSW 2052 Australia; 3 School of Biological, Earth and Environmental Sciences, University of New South Wales, Sydney, NSW 2052 Australia; 4 Department of Genomic and Applied Microbiology, Georg-August-University Göttingen, Grisebachstr. 8, 37077 Göttingen, Germany

**Keywords:** Metagenomics, Horizontal gene transfer, HGT identification, Bioinformatics

## Abstract

**Background:**

Metagenomic datasets provide an opportunity to study horizontal gene transfer (HGT) on the level of a microbial community. However, current HGT detection methods cannot be applied to community-level datasets or require reference genomes. Here, we present MetaCHIP, a pipeline for reference-independent HGT identification at the community level.

**Results:**

Assessment of MetaCHIP’s performance on simulated datasets revealed that it can predict HGTs with various degrees of genetic divergence from metagenomic datasets. The results also indicated that the detection of very recent gene transfers (i.e. those with low levels of genetic divergence) from metagenomics datasets is largely affected by the read assembly step. Comparison of MetaCHIP with a previous analysis on soil bacteria showed a high level of consistency for the prediction of recent HGTs and revealed a large number of additional non-recent gene transfers, which can provide new biological and ecological insight. Assessment of MetaCHIP’s performance on real metagenomic datasets confirmed the role of HGT in the spread of genes related to antibiotic resistance in the human gut microbiome. Further testing also showed that functions related to energy production and conversion as well as carbohydrate transport and metabolism are frequently transferred among free-living microorganisms.

**Conclusion:**

MetaCHIP provides an opportunity to study HGTs among members of a microbial community and therefore has several applications in the field of microbial ecology and evolution. MetaCHIP is implemented in Python and freely available at https://github.com/songweizhi/MetaCHIP.

**Electronic supplementary material:**

The online version of this article (10.1186/s40168-019-0649-y) contains supplementary material, which is available to authorized users.

## Background

Genome reconstruction (binning) of uncultured microorganisms has recently become feasible due to the comprehensive sequencing of microbial community DNA (metagenomic DNA) and novel computational approaches [1–3]. The reconstructed genome bins have provided new insights into the biochemistry, physiology and adaptation of previously uncharacterized microbial groups [4–8]. Moreover, they offer the opportunity to study horizontal gene transfer (HGT) within communities of uncultured microorganisms.

HGT, the transmission of genetic information between organisms, is thought to be an important driver of microbial evolution and adaptation, including the development of antibiotic resistance and virulence [9, 10]. Several bioinformatics tools have been developed using a range of algorithms and features to identify HGTs. For example, GIST [11] and IslandViewer [12] utilize the compositional features of genome sequences to predict HGT events, while DarkHorse [13] and HGTector [14] use the sequence similarities (best matches) for HGT prediction. Explicit phylogenetic approaches are employed by Ranger-DTL [15] and AnGST [16], which predict HGTs through the reconciliation of gene trees with corresponding species trees.

However, current HGT detection methods cannot be applied to the entire communities or require reference genomes. For example, HGTector [14] can only detect HGTs from members in a defined distal group to defined self-group members, which limits its application to predict HGTs among all members within a microbial community, while DarkHorse [13] requires suitable reference genomes to predict HGTs, which are often not available for uncultured microorganisms.

We therefore developed here MetaCHIP (“Meta” for “metagenomics”, “CHIP” for “Community-level HGT Identification Pipeline”), a pipeline for the reference-independent and community-level identification of HGTs. Our analysis of simulated and real data showed that MetaCHIP can detect HGTs from communities with a high degree of confidence and to give new biological and ecological insights.

## Methods

**Fig. 1:**
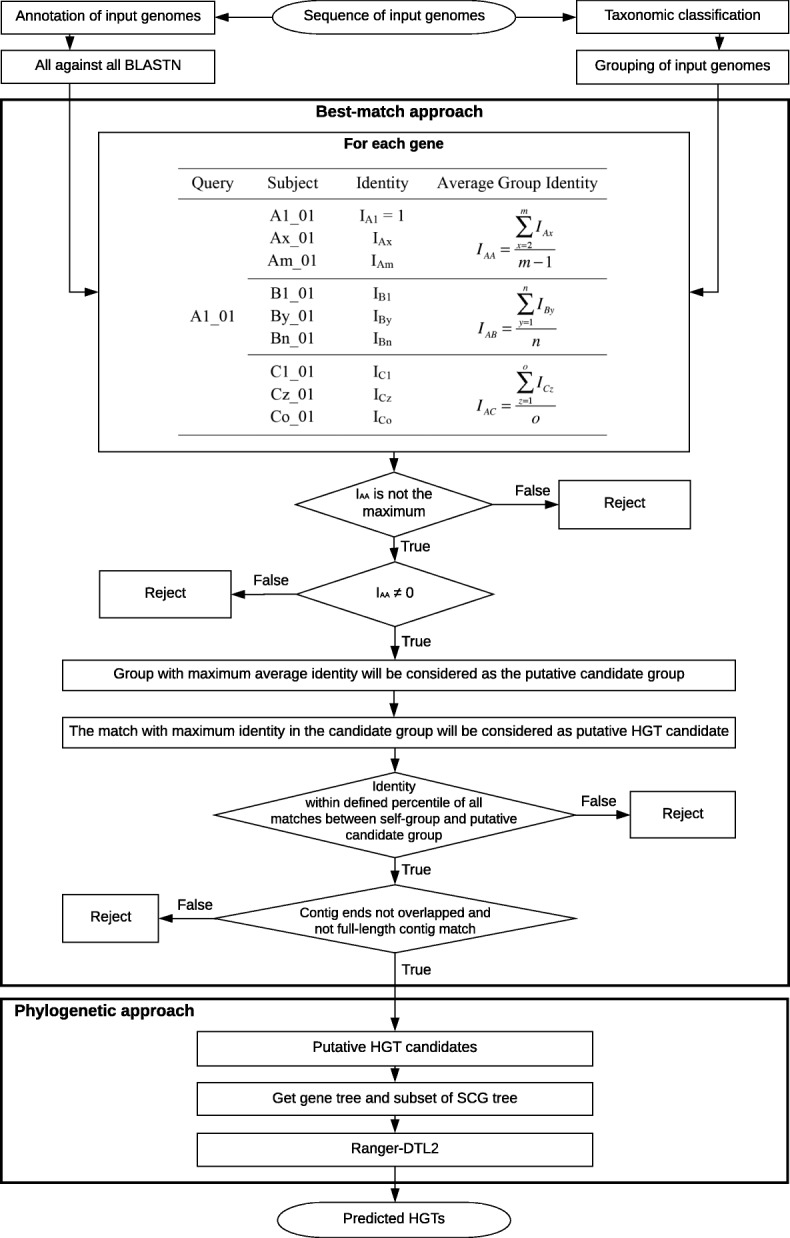
Workflow of MetaCHIP

The workflow of MetaCHIP is presented in Fig. 1. MetaCHIP uses both best-match and phylogenetic approaches for HGT detection (see above). Its inputs are the sequence files of a set of genomes or genome bins derived from metagenomic data as well as their taxonomic classifications. The recently developed GTDB-Tk tool [17], which is based on the phylogenetically calibrated Genome Taxonomy Database (GTDB) [18], is recommended for the taxonomic classification of input genomes. Input genomes are initially grouped by MetaCHIP according to their taxonomic classifications at user-specified rank (e.g. class, order, family or genus).

### Best-match approach

Open reading frames (ORFs) are predicted from input genomes with Prodigal v2.6.3 [19], and an all-against-all BLASTN [20] search is performed among all predicted ORFs. The BLASTN results are first filtered with user-defined alignment length (e.g. 200 bp) and coverage cut-offs (e.g. 75%). The filtered matches are then compared between groups of genomes using the following steps. Here, we suppose all input genomes are divided into three groups (A, B and C), with individual genomes referred to as Ax, By and Cz, respectively (Fig. 1). Genes from each genome are represented as Ax_N, By_N and Cz_N. Take gene A1_01 as an example, the number of its BLASTN matches from groups A, B and C is m, n and o, respectively, with their corresponding identities being I_Ax_, I_By_ and I_Cz_. The average identities of the matches from each group are I_AA_, I_AB_ and I_AC_, respectively (Fig. 1). The following analyses are then performed for each gene (here as an example with A1_01):

1. If I_AA_ is the maximum, which means all its best matches are coming from the self-group, then gene A1_01 is not a candidate for HGT.
2. If I_AA_ = 0 (that is, only the self-match was found from group A), then all BLASTN matches from other groups will be ignored. This is because, if the non-self-group subject with maximum identity was considered a HGT candidate, then it is very likely to be a false positive due to the lack of self-group matches.
3. If I_AA_ ≠ 0 and I_AA_ is not the maximum, then the non-self-group with maximum average identity (e.g. I_AB_ or I_AC_) will be considered as a putative candidate group for HGT.
4. The BLASTN match with maximum identity in the candidate group will be considered the putative HGT candidate.
5. Identity distribution of all genes between the self-group and the putative candidate group is summarized. The identity cut-off corresponding to pre-defined percentile (e.g. the highest 10%) is calculated. Only putative HGT candidates which have identities higher than this cut-off will be further considered.

### Analysis of regions flanking putative HGTs

Assembly algorithm based on DeBruijn graphs (e.g. SOAP [21], Velvet [22], SPAdes [23], IDBA [24]) will produce “bubbles” for sequence regions with sequencing error, but high similarity [25]. The resolution of such bubbles may produce two contigs with overlapping sequences at the end of the contigs. This duplication could be falsely considered in the HGT analysis, and to avoid this, putative HGT candidates located at contigs’ end with high similarity (> 95%) are disregarded. In addition, putative HGT candidates located on contigs, which had 95% of their full-length matching with a longer contig, were disregarded, as these contigs are likely artificial duplicates of the assembly process.

**Fig. 2:**
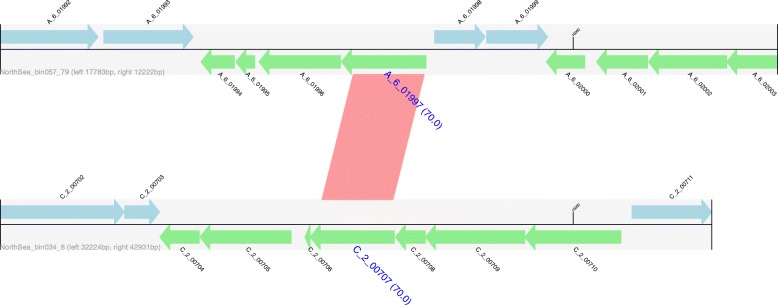
Example output for the flanking regions of an identified HGTs. Genes encoded on the forward strand are displayed in light blue, and genes coded on the reverse strand are displayed in light green. The names of genes predicted to be HGT are highlighted in blue, large font with pairwise identity given in parentheses. Contig names are provided at the left bottom of the sequence tracks, and numbers following the contig name refer to the distances between the gene subject to HGT and either the left or right end of the contig. Red bars show similarities of the matched regions between the contigs based on BLASTN results

To further corroborate the predicted HGT candidates, their flanking sequences within user-defined length (e.g. 10 kbp) are extracted from the annotation files. A pairwise BLASTN is performed between each pair of flanking regions. Plots for the genomic regions are generated with GenomeDiagram [26] and provided for visual inspection (Fig. 2).

### Phylogenetic approach

A phylogenetic approach is used to further corroborate the results given by the best-match approach and to provide information on the direction of gene flow. For each pair of genes, which were identified as putative HGT by the best-match approach, a protein tree is generated using the genes used for the HGT analysis in the best-match approach and all orthologs from the two groups, from which the paired genes came from. Amino acid sequences are aligned with MAFFT v7.310 [27] and followed by the removal of columns represented by < 50% of proteins and/or with an amino acid consensus of < 50%. A protein tree is then constructed using FastTree v2.1.10 [28] with default parameters.

A “species” tree is then generated to compare to the gene tree. As the 16S rRNA gene, which is the most commonly used phylogenetic and taxonomic marker of bacterial and archaeal organisms, is often missing in genome bins [29–31], we build a phylogenetic tree for all input genomes using the protein sequences of 43 universal single-copy genes (SCGs) used by CheckM [32]. Predicted protein sequences for the input genomes are searched for the PFAM v31.0 [33] and TIGRFAM v14.0 [34] hmm profiles of these SCG proteins using HMMER v3.1b2 [35]. Protein sequences for each hmm profile are then individually aligned using HMMER and concatenated into a multiple sequence alignment (MSA). Columns represented by < 50% of genomes and/or with an amino acid consensus < 25% are removed, and a phylogenetic tree is built using FastTree [28]. A subtree, which includes only the genomes relevant to the particular genes analysed is extracted with preserved branch length using ETE v3.1.1 [36]. The reconciliation between each pair of protein tree and “species” subtree is performed using Ranger-DTL v2.0 with dated mode. Briefly, Ranger-DTL predicts HGTs by performing a duplication-transfer-loss (DTL) reconciliation between a protein family phylogeny and its corresponding organismal phylogeny [15].

To assess how reliable SCG protein trees are to reconstruct organismal phylogenies from partial genome bins, we selected 20 alpha- and beta-proteobacterial genomes (see below) and divided each of them into 100 contigs with equal length. Next, 20, 40, 60 and 80 contigs were randomly selected to represent genome bins with 20, 40, 60 and 80% completeness, respectively. The similarities between the SCG protein trees with these different levels of completeness and the tree based on 16S rRNA gene sequences were then assessed by Mantel tests [37].

### Assessment of MetaCHIP on simulated datasets

MetaCHIP’s performance was first assessed on simulated datasets at different taxonomic levels. To assess its performance at a low taxonomic level, ten genomes from species of the genus *Sphingobium* (donor group) and *Sphingomonas* (recipient group) within the family *Sphingomonadaceae* were selected (see Additional file 1: Table S1), while for class level transfers, ten alphaproteobacterial (donor group) and betaproteobacterial (recipient group) genomes were chosen (see Additional file 1: Table S2). Ten genes (with at least two orthologs in the recipient group) from each of the ten donor genomes were selected and randomly transferred into the ten recipient genomes with different levels of genetic divergence (0, 5, 10, 15, 20, 25 and 30%) using HgtSIM [38]. The six-frame stop codon sequence “TAGATGAGTGATTAGTTAGTTA” was added to the two ends of transferred genes to facilitate correct gene prediction. This process was bootstrapped ten times, and donor and mutated recipient genomes from each bootstrap were used directly as inputs into MetaCHIP to assess its performance at class and genus levels.

Sequencing reads were also simulated from the ten alphaproteobacterial and ten mutated betaproteobacterial genomes for each level of genetic divergence from one of the ten bootstraps. Sequencing reads for each level of genetic divergence were simulated three times with different abundance profiles (Additional file 1: Table S3) using GemSIM [39].

As the reconstruction of genes involved in HGT are highly affected by sequencing depth or the assembler used [38], 3, 6, 9 and 12 million reads, corresponding to an average coverage of approximately 6, 11, 17 and 23×, were simulated for each level of genetic divergence. The paired-end reads were quality filtered using Trimmomatic v0.36 [40] with a quality cut-off of 20 and a sliding window of 6 bp. Reads from the 3 replicates were combined and then assembled with IDBA_UD v1.1.1 [24] or metaSPAdes v3.9.0 [23], and contigs were filtered with a length cut-off of 2500 bp. A gene transfer was considered to be reconstructed during the assembly process, if at least 1 of the gene’s 2 flanking regions was > 1 kbp and the flanking region matched the recipient genome [38]. The existence of gene transfers in the filtered contigs was analysed by performing a pairwise BLASTN between the transferred genes and the contigs for each level of genetic divergence. The BLASTN results were then filtered with an identity cut-off of > 98% and a coverage cut-off of > 98% for the transferred genes.

Metagenome binning was performed with MetaBAT v0.32.5 [1] and MyCC v2017 [2], and the results were refined with Binning_refiner v1.2 [41]. Bin completeness and contamination were assessed with CheckM v0.9.7 [32]. The correlations between the genome bins and the reference genomes were obtained by running pairwise BLASTN searches. The correlations between MetaCHIP-predicted HGTs and the known simulated gene transfers were determined by running pairwise BLASTN searches with identity and coverage cut-off of > 98%.

### Assessment of MetaCHIP on a dataset with previously described HGTs

MetaCHIP’s performance was also assessed on 2094 full bacterial genomes, which were previously analysed for HGTs using blocks of nearly identical DNA (> 99% identity, over 500 bp) in distantly related genomes (16S rRNA gene similarity less than 97%) [42]. The 2094 bacterial genomes were downloaded from the NCBI RefSeq database, and their taxonomy was determined using GTDB-Tk v0.1.6 [17]. HGT events were then analysed with MetaCHIP at the genus level. BLASTN search with a 100% identity and coverage cut-off were used to compare MetaCHIP-predicted HGTs with previously identified, transferred DNA blocks. COG annotation of predicted HGTs was performed by running RPS-BLAST [20] against the COG database [43].

### Assessment of MetaCHIP on real metagenomic dataset

Genome bins derived from metagenomic datasets for microbiomes from human guts [1, 44] and seawater samples taken in the North Sea [45] were used to assess the performance of MetaCHIP on real metagenomic datasets. For the human gut dataset, genome bins previously produced by MetaBAT [1] were used directly here after removing the contigs shorter than 2000 bp. For the North Sea dataset, all sequencing reads were quality filtered with Trimmomatic as previously described [45] and assembled using metaSPAdes v3.9.1. Binning was performed as described above. CheckM v0.9.7 was subsequently used to assess the quality of genome bins. The SCG protein tree of these bins and COG annotation of predicted HGTs were performed as described above, and antibiotic resistance-related COGs were retrieved from the Antibiotic Resistance Genes Database (ARDB; April 2018) [46].

## Results and discussion

### Performance on simulated datasets

**Fig. 3:**
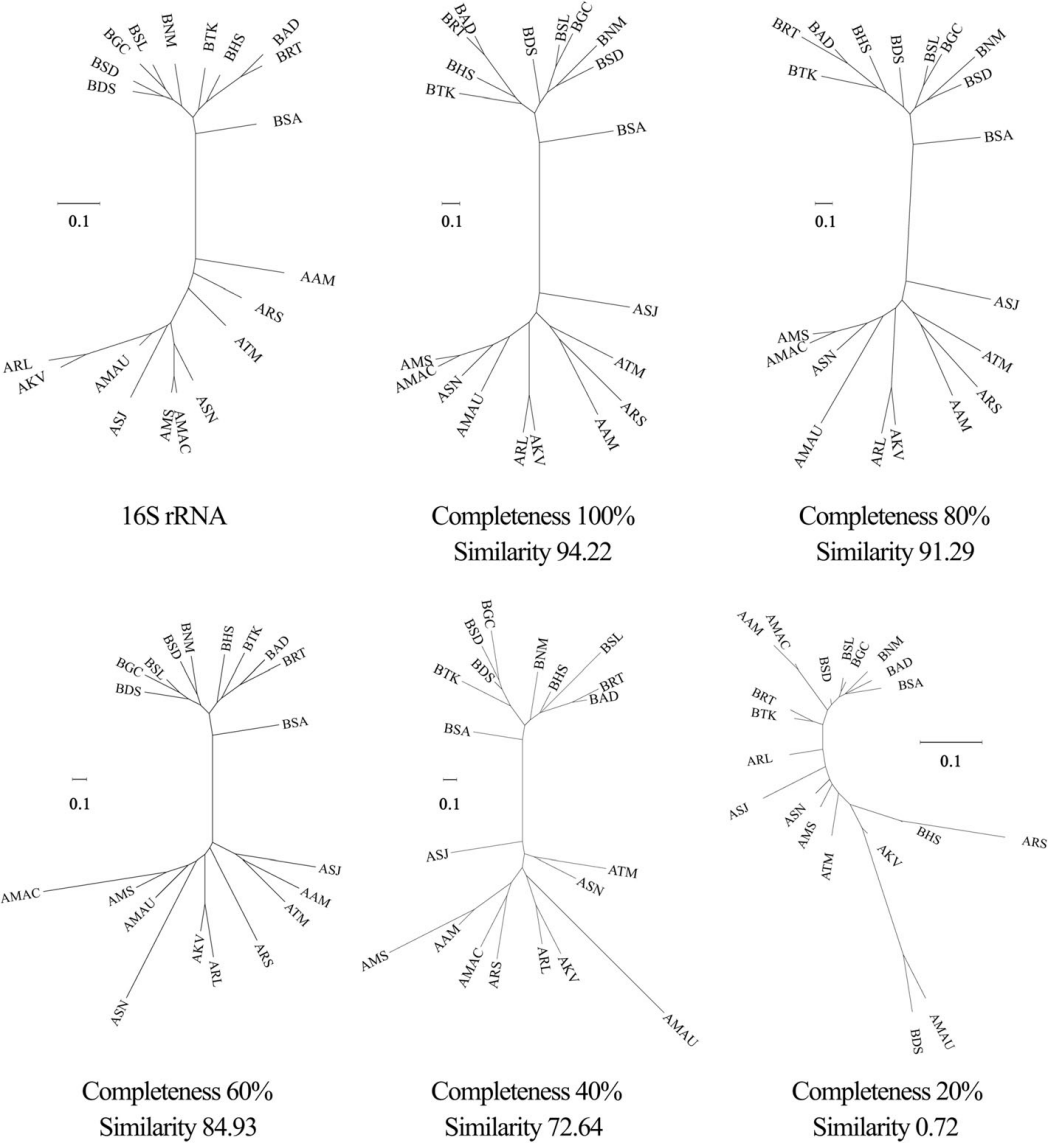
The similarity between the tree based on 16S rRNA gene sequences and the SCG protein trees with different level of genome completeness. Similarities were assessed by Mantel tests

MetaCHIP requires a SCG protein tree of all input genomes for the phylogenetic approach. We therefore first assessed how reliable the reconstruction of a SCG-based phylogeny is for incomplete genome bins. The results showed a high degree of congruence between the SCG protein trees and the tree based on 16S rRNA gene sequences for genome bins with completeness higher than 40% (Fig. 3). This value is thus suggested for the completeness cut-off for genome bins used as input for MetaCHIP.

**Fig. 4:**
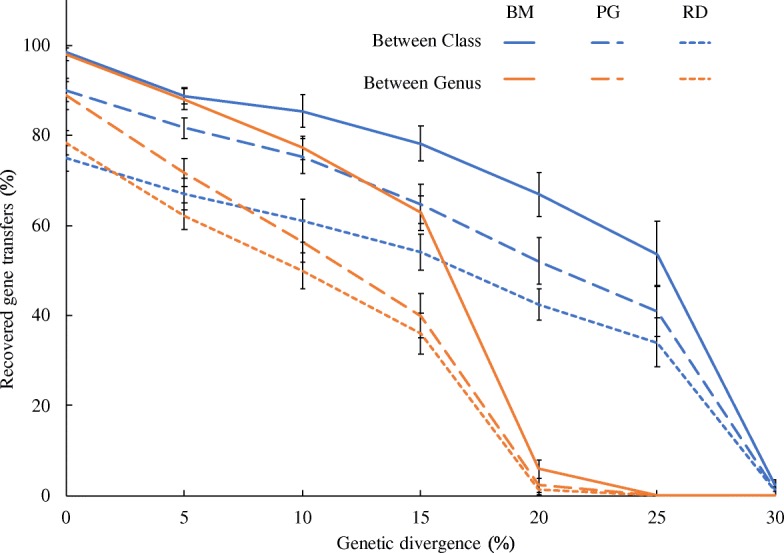
MetaCHIP’s performance on the recovery of HGTs introduced between genomes at class and genus levels. “BM” and “PG” refer to the recovered gene transfers after the “best-match approach” and “phylogenetic approach”, respectively. “RD” shows the recovery of predictions with the “right direction” of gene flow

MetaCHIP’s performance was first assessed by introducing defined HGTs in genomes at genus and class levels. MetaCHIP had a high recovery rate of artificially introduced HGTs up to the levels of genetic divergence of 10% at both taxonomic levels (Fig. 4). A steady decline in recovery was observed with higher levels of genetic divergence. Detection of between-class HGTs became unsuccessful at 30% divergence, while at the genus level, the detection threshold was reached at around 20% divergence. This performance is consistent with previous findings and algorithms that showed the difficulties of detecting HGT between closely related taxa (e.g. genera of the same family) [47, 48]. Nevertheless, no less than 40% of between-genera HGTs with genetic divergence less than 15% can be detected by MetaCHIP. The phylogenetic analysis predicted the correct directions of gene flow in more than 81% of cases for transfers between classes at all divergence levels and in more than 86% of cases between genera with genetic divergence less than 15% (Fig. 4).

**Fig. 5:**
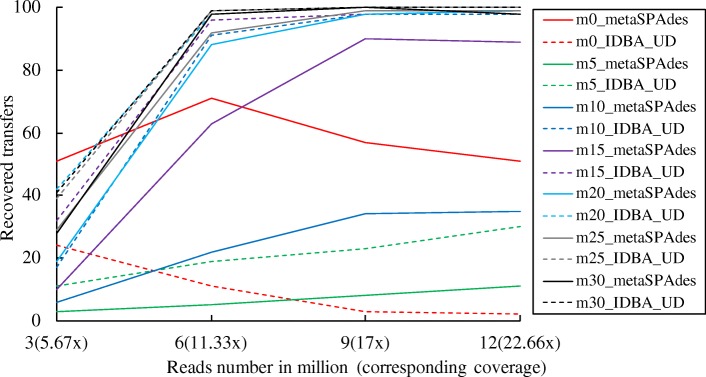
The effect of sequencing depth on the recovery of introduced gene transfers with different assemblers and different levels of genetic divergence

We next evaluated how different assemblers and sequencing depths influence the recovery of class-level HGTs with different levels of genetic divergence. We also introduced realistic sequencing errors into the read dataset before assembly. When no mutation was introduced to the transferred genes, more transferred genes were recovered by metaSPAdes than with IDBA_UD. For 5% genetic divergence, both assemblers performed overall quite poorly in terms of the recovery rate of introduced gene transfers, but IDBA_UD had generally a better recovery rate than metaSPAdes. IDBA_UD showed also better recovery for HGTs with divergence levels between 10 and 30% (Fig. 5). MetaSPAdes was therefore used for the assembly of metagenomic reads with no genetic divergence, while IDBA_UD was selected for datasets with the other levels of genetic divergence. For gene transfers with no genetic divergence, the recovery rate for metaSPAdes assemblies was the highest with a sequencing depth of 11.33×, beyond which it declined. For the 5% genetic divergence, the best recovery from the IDBA_UD assemblies was at sequencing depths of 11.3× or greater (Fig. 5). As a compromise for the non-linear behaviour of recovery rates, a sequencing depth of 17× (9 million reads) was selected for all subsequent simulations.

Based on these choices of coverage and assembler, we next binned the genomes from the simulated datasets. The precision (defined as how pure a bin is) and recall (defined as how complete a bin is) of the genome bins for all divergence groups were calculated with evaluate.py from the MyCC package [2]. The results showed that their overall precision and recall were not lower than 99.73% and 89.49%, respectively (Table 1).

**Table 1:** The quality of refined genome bins reconstructed from simulated metagenomic datasets at different level of genetic divergence of introduced HGTs

Genetic divergence (%)	0	5	10	15	20	25	30
Precision (%)	99.73	99.96	99.95	99.97	99.93	99.97	100.00
Recall (%)	89.49	93.20	95.92	96.46	95.41	96.45	96.35

**Fig. 6:**
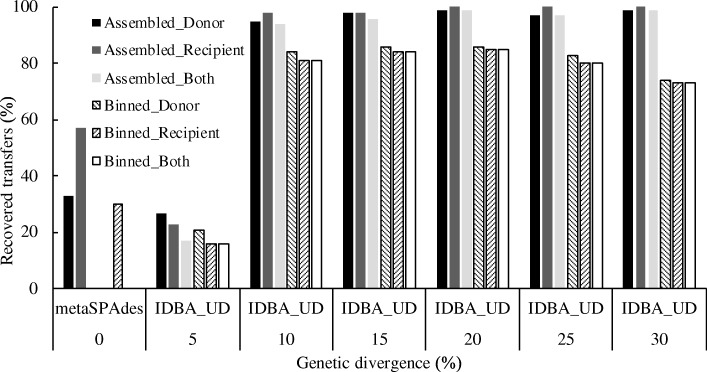
The percentage of recovered gene transfers during assembly and binning

We next investigated the presence of introduced gene transfers in these genome bins. For 0% genetic divergence, 30% of introduced gene transfers were identified in the genome bins and all of them were found in the recipient genomes. For the levels of genetic divergence greater than 5%, no less than 73.7% of transferred gene copies were found in both the donor and recipient genome bins (Fig. 6).

**Fig. 7:**
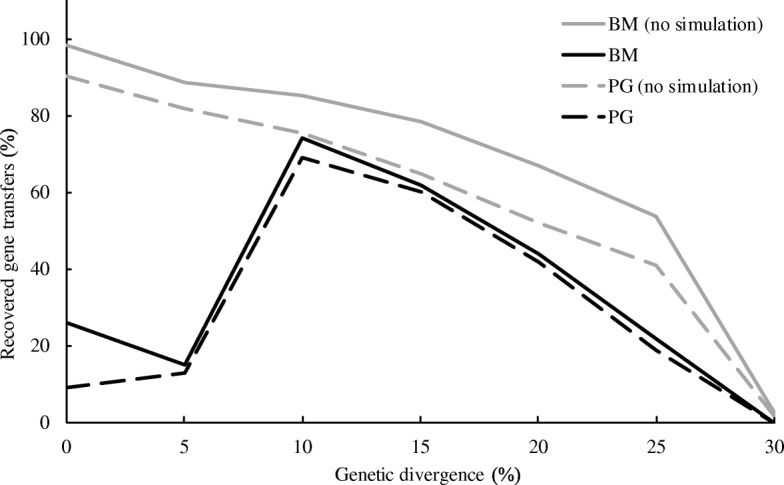
The percentage of recovered gene transfers by MetaCHIP after assembly of simulated reads and binning of genomes (simulation). For comparison, the results from original genomes (no reads simulation) are also shown and are the same as in Fig. 4

By applying MetaCHIP to the genome bins, 26% of the 100 introduced gene transfers were recovered by the best-match approach for the 0% genetic divergence and nine of them were validated by the phylogenetic approach (Fig. 7), which accounts for 86.7% and 30%, respectively, of the gene transfers that actually exist in the genome bins. For a 5% genetic divergence, 93.8% of introduced gene transfers that were found in the bins were also identified by the best-match approach and 81.3% of them were validated by the phylogenetic approach. The best recovery rates were obtained when the genetic divergence is 10%, where at least 74% of introduced gene transfers were recovered by the best-match approach and 69% of them were validated by the phylogenetic approach, which accounted for 91.4% and 85.2% of all binned gene transfers, respectively. A steady decline in the ability of MetaCHIP to detect HGT was also observed with higher genetic divergence (Fig. 7).

### Performance on dataset with previously described HGTs

**Fig. 8:**
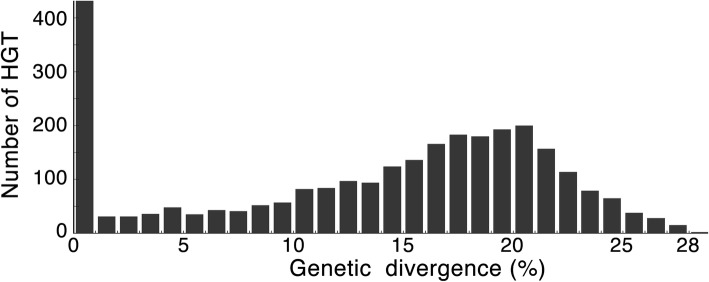
Genetic divergence of MetaCHIP identified HGTs from the 368 genomes of soil isolates

We next benchmarked MetaCHIP’s performance against a previous large-scale study that analysed HGT in 2235 bacterial genomes [42]. Only 2094 of the genome from [42] were available for download from the NCBI RefSeq database (Additional file 2), and they were subsequently grouped by MetaCHIP into 664 genera. MetaCHIP identified 10,255 HGTs (Additional files 3 and 4), of which 2694 (26.3%) showed a genetic divergence less than 1%, i.e. represent recent transfers (Fig. 8). We compared MetaCHIP’s prediction with previously described HGTs only for the soil isolates, as metadata for the other isolate types were missing or incomplete (see Supplementary Table S5 from [42]). Four hundred thirty-three of the 2694 recent gene transfers fulfil the criteria that they were between genomes with 16S rRNA gene similarity less than 97% (a filter implemented in [42]). The previous analysis identified 368 HGTs with no more than 1% genetic divergence involving soil isolates, and 248 (77.2%) of them overlap with MetaCHIP’s predictions, which showed relatively high consistency between the two approaches.

**Fig. 9:**
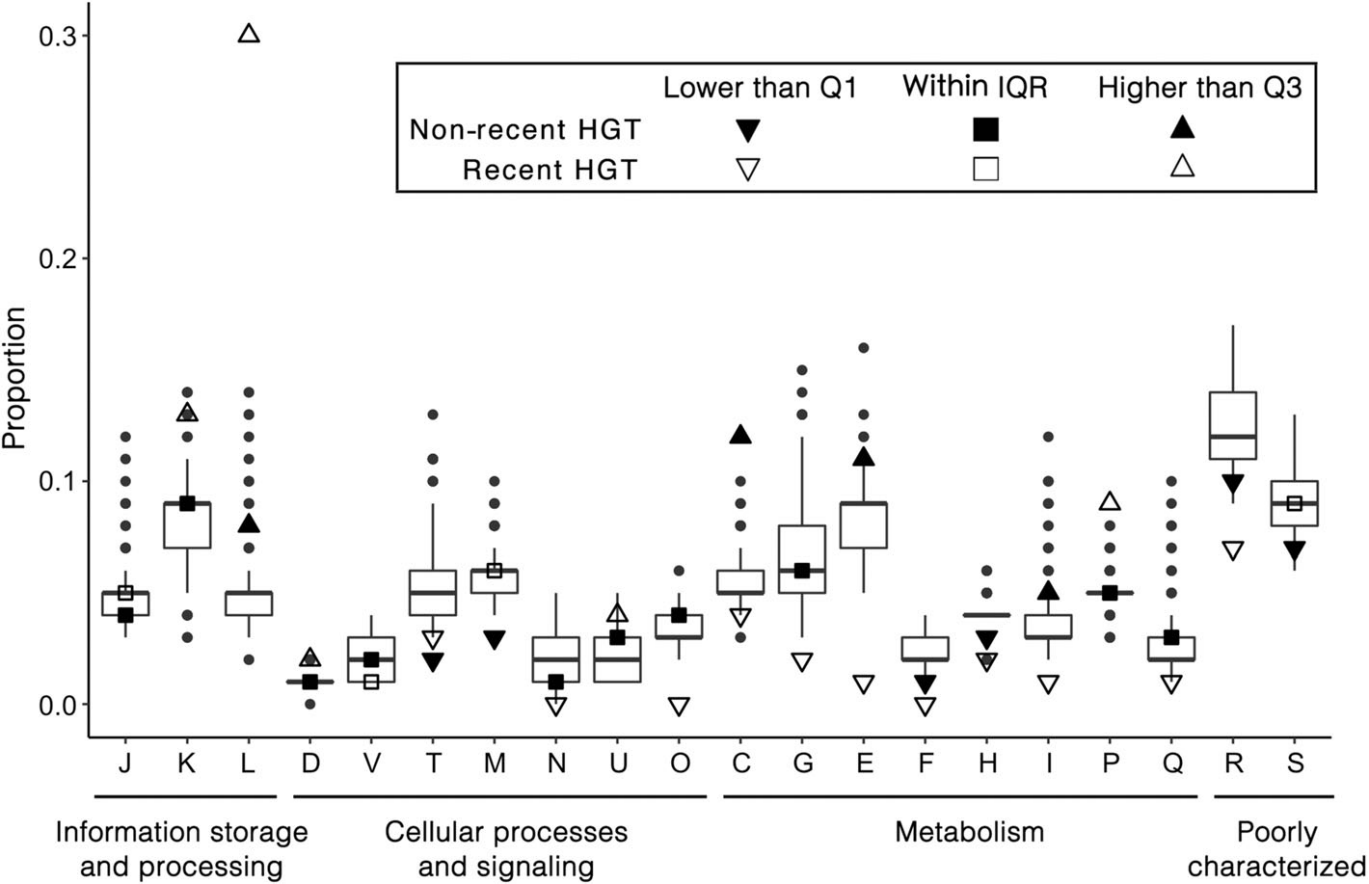
Relative proportion of COG functional categories for the 2094 genomes as well as MetaCHIP predicted recent (with genetic divergence ≤ 1%) and non-recent (with genetic divergence > 1%) HGTs. The boxes in the plot are bound by the 25% to 75% quartile proportions with the thick line being the median value. Q1, Q3 and IQR refer to the 25%, 75% and interquartile range, respectively. The upper whisker refers to the largest observation less than or equal to upper Q3 + 1.5 × IQR, while the lower whisker refers to the smallest observation greater than or equal to Q1 − 1.5 × IQR. The letters on the *x*-axis indicate COG categories: C (energy production and conversion), D (cell cycle control, cell division and chromosome partitioning), E (amino acid transport and metabolism), F (nucleotide transport and metabolism), G (carbohydrate transport and metabolism), H (coenzyme transport and metabolism), I (lipid transport and metabolism), J (translation, ribosomal structure and biogenesis), K (transcription), L (replication, recombination and repair), M (cell wall/membrane/envelope biogenesis), N (cell motility), O (posttranslational modification, protein turnover and chaperones), P (inorganic ion transport and metabolism), Q (secondary metabolites biosynthesis, transport and catabolism), R (general function prediction only), S (function unknown), T (signal transduction mechanisms), U (intracellular trafficking, secretion and vesicular transport) and V (defence mechanisms)

Besides the 433 recent HGTs, MetaCHIP also identified 2414 gene transfers with genetic divergence higher than 1% involving the genome of soil isolates (Fig. 8). Recent (i.e. genetic divergence ≤ 1%) and non-recent (i.e. genetic divergence > 1%) HGTs predicted by MetaCHIP and all genes for the 368 soil genomes were then annotated using the COG system. COG categories were considered to be enriched in the HGT dataset if their proportion was above the 75% percentile of the relative abundance across all input genomes. The results revealed that COG categories enriched for recent HGTs are different to those enriched in the non-recent HGTs. For example, COG categories C (energy production and conversion), E (amino acid transport and metabolism), I (lipid transport and metabolism) and L (replication, recombination and repair) were only enriched in the non-recent HGTs, while categories K (transcription), P (inorganic ion transport and metabolism) and U (intracellular trafficking, secretion and vesicular transport) were enriched in recent HGTs (Fig. 9). This observation was missed by the previous analysis [42] and shows that MetaCHIP can provide new biological and ecological insights into the HGT of microbial communities.

### Performance on real metagenomic datasets

**Fig. 10:**
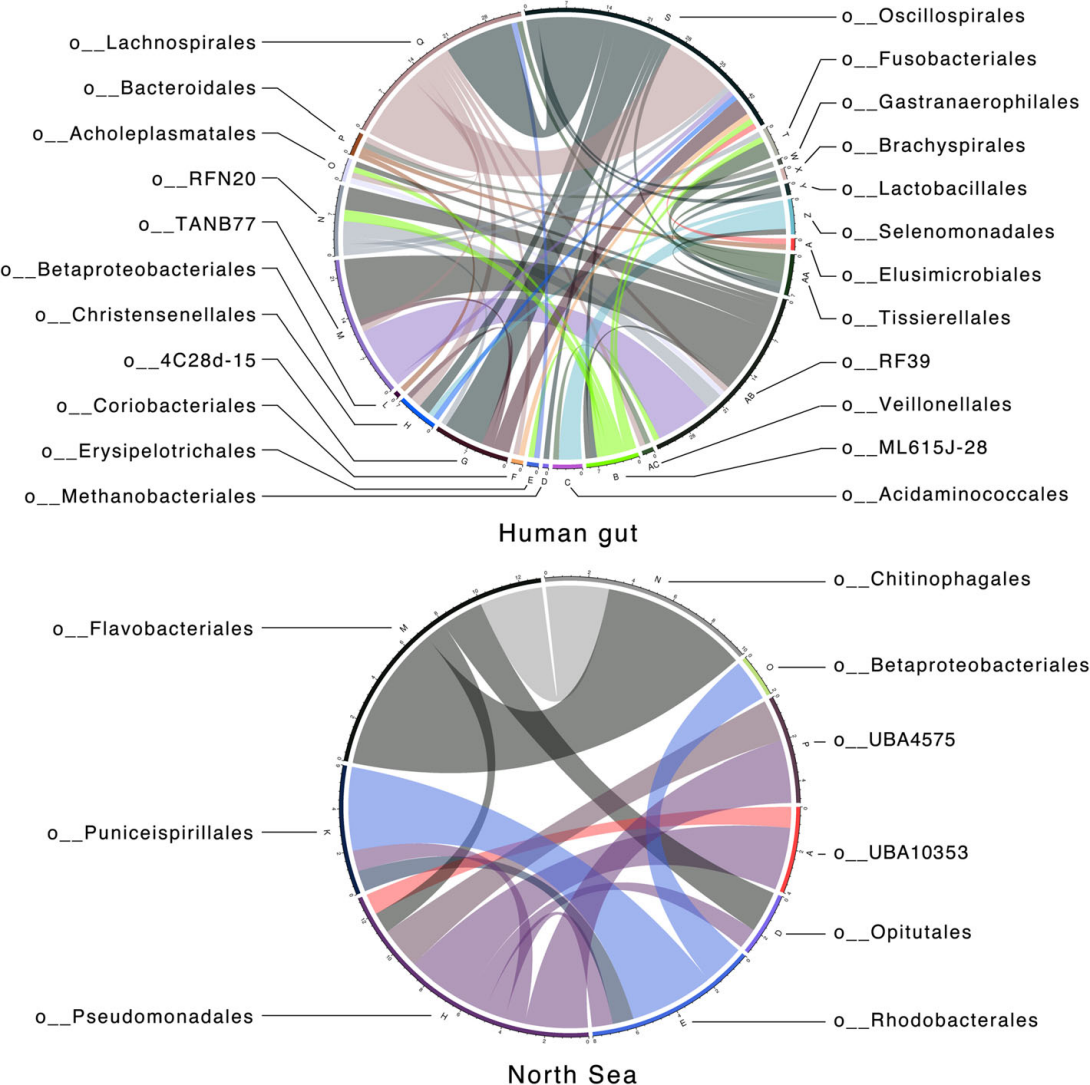
Predicted gene flow within the human gut and North Sea microbial communities. Bands connect donors and recipients, with the width of the band correlating to the number of HGTs and the colour corresponding to the donors

Finally, we assessed MetaCHIP’s performance on two real metagenomic datasets: one for free-living seawater microorganisms in the North Sea [49] and the other for the human gut microbiome [44]. For the metagenomic dataset of seawater microorganisms, sequence assembly with metaSPAdes generated 315.33 Mbp of contiguous sequences ≥ 2500 bp (35,190 contigs) and 69 genome bins were obtained, of which 37 had no contamination as detected with CheckM and completenesses higher than 40%. For the 1634 genome bins obtained from the human gut dataset described in [44], 138 were estimated to be contamination-free and more than 40% complete. The taxonomy of qualified genome bins was determined with GTDB-Tk (Additional file 5). The human gut and the seawater bins were taxonomically grouped into 29 and 16 orders, respectively (Additional file 1: Figure S1). The best-match approach detected 560 gene transfers for the human gut genome bins, and of which 113 were also found by the phylogenetic approach. For the seawater dataset, 121 and 32 gene transfers were detected by the two approaches, respectively. The direction of predicted gene flows within the two communities was shown in Fig. 10. Not surprisingly, the number of HGT detected in any given group is proportional to the number of genome bins it contained (Additional file 1: Figure S1 and Fig. 10). One exception however is the order *Bacteroidales* from the human gut dataset, where only four HGTs were detected in its 30 genome bins. High rates of HGT within the order *Bacteroidales* have been previously described [50], but our results indicate that this does not apply to HGTs of this order with other taxonomic groups. A genome bin of the *Chitinophagales* from the North Sea dataset was also found to have all its 10 HGTs with genomes from the order *Flavobacteriales* (Fig. 10), which is consistent with previous observation that HGT is more likely to occur between those two closely related taxa [51].

**Fig. 11:**
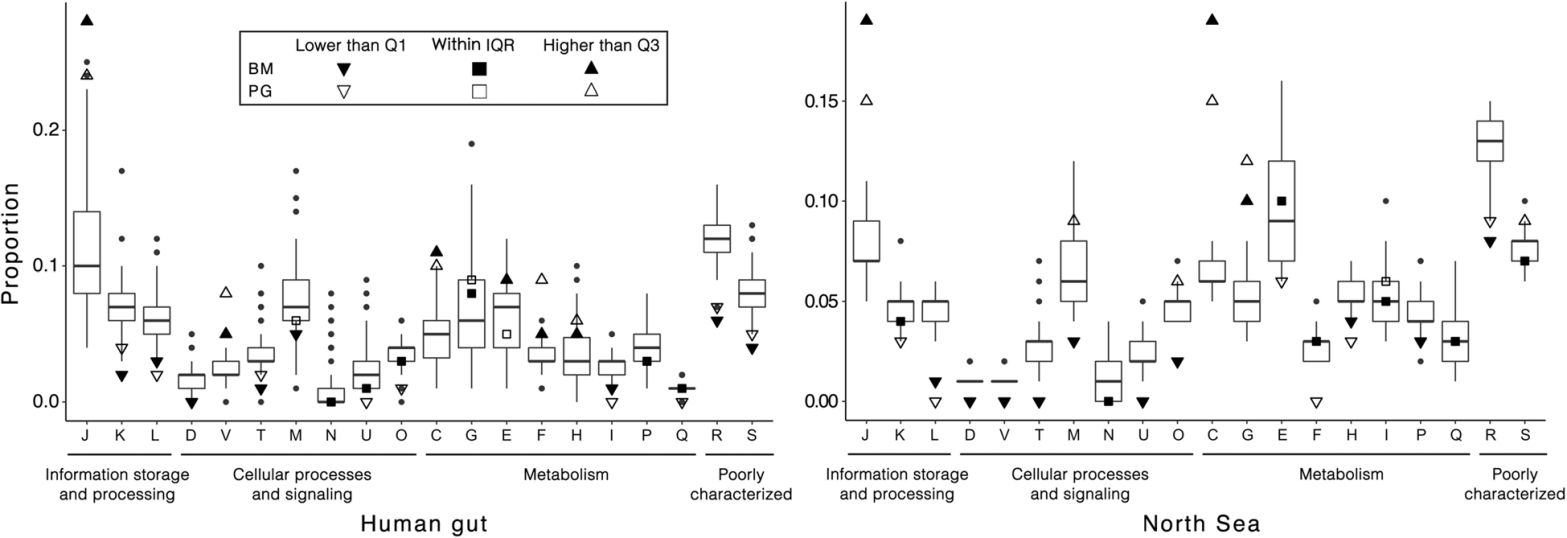
Relative proportion of COG functional categories for the input genome bins and predicted HGTs from human gut bins. The boxes in the plot are bound by the 25% to 75% quartile proportions with the thick line being the median value. Q1, Q3 and IQR refer to the 25%, 75% and interquartile range, respectively. The upper whisker refers to the largest observation less than or equal to upper Q3 + 1.5 × IQR, while the lower whisker refers to the smallest observation greater than or equal to Q1 − 1.5 × IQR. COG categories indicated by letters on the x-axis are the same as in Fig. 9

We next performed a functional annotation of the genes identified in the HGT analysis based on the COG system as described above. The results for the human gut dataset showed that genes subject to HGT were enriched for the COG categories of defence mechanisms (V); energy production and conversion (C); translation, ribosomal structure and biogenesis (J); and nucleotide transport and metabolism (F) (Fig. 11). The enrichment of defence mechanisms (V) was mainly due to 15 HGTs involving genes with functions related to ABC-type multidrug (COG1131) and antimicrobial peptide (COG1136) transport systems. This observation is consistent with previous observations and proposals that HGT is a dominant factor for the spread of AR in the human gut microbiota [52–54]. For example, a variety of genes for ABC-type multidrug transport systems have been previously found to be often associated with transposable elements in the gut microbiomes, and this was postulated to facilitate their horizontal transfer [55]. COG categories preferentially subject to HGT between the free-living microorganisms in the North Sea include energy production and conversion (C), carbohydrate transport and metabolism (G) and translation, ribosomal structure and biogenesis (J) (Fig. 11). This observation is similar to a recent study on HGTs among all available complete genomes for free-living *Archaea* and *Bacteria*, where transferred genes most frequently also belonged to COG categories C and G [56].

More than 40% of identified HGTs for the human gut and the seawater bins had a genetic divergence of 25 ± 2.5% (Table 2). The best-match and phylogenetic approaches only detected 19% and 4%, respectively, of introduced gene transfers with this level of genetic divergence in the simulated datasets (Fig. 7), and hence, we predict that the actual numbers of HGTs that occurred in the community are likely to be underestimated here. Interestingly, only one transfer with genetic divergence at less than 15% divergence was detected, for which we generally found a high recovery rate in our simulations (Fig. 7). This may indicate that HGT in these microbial communities does not involve a large number of recent transfers or that the actual donors were not recovered in the genome bins due to assembler limitations [38] or the removal of low-quality genome bins.

**Table 2:** Genetic divergence of HGT identified by MetaCHIP from the human gut and North Sea datasets

Dataset	Approach	Genetic divergence (± 2.5%)						
		0	5	10	15	20	25	30
Human gut	Best-match	0	0	0	1	79	406	74
	Phylogenetic	0	0	0	0	18	78	17
North Sea	Best-match	0	0	0	0	8	78	35
	Phylogenetic	0	0	0	0	4	14	14

## Conclusion

Our development and tests of MetaCHIP showed that the tool can detect HGTs with various degree of genetic divergence from microbial community data, but that prediction efficiency is affected by several factors. First, as transferred genes will undergo mutations in their new genomic contexts, their detections will become difficult when the similarities between the donor and the recipient genes fall below certain levels (Fig. 3) [57]. Second, the detection of recent gene transfers (i.e. those with very little variation between donor and recipient) is largely affected by technical limitations of metagenomic analyses. As current sequencing technologies and assemblers often failed to assemble long regions with high sequence similarity [38, 58], recent HGTs will not be captured effectively in the genomic context of the donor and recipient (Fig. 5). This problem might be addressed in the near future by long-read sequencing technologies, such as PacBio’s sequencing platform [59], when applied to metagenomic samples. Third, the successful detection of HGT from metagenomic dataset requires the reliable reconstruction of the organismal genomes, in particular, through genome binning, as mis-binned sequences (contamination) may introduce false positives in the HGT analysis, and reliable organismal tree for phylogeny-based prediction of HGTs requires a certain degree of genome completeness (e.g. 40%) (Fig. 3). Improvement of genome binning accuracy can be achieved either by incorporating more biological samples [1] or by combining the binning results from multiple binning programmes [41], while the completeness of genome bins can be improved with higher sequencing depth. Despite these limitations, our analysis of simulated and real data with MetaCHIP shows that HGTs can be detected from microbial community data with a high degree of confidence to give new biological and ecological insights. However, the absolute numbers of HGTs that occur in the community might be underestimated given the limitations outlined above.

## Additional files

Additional file 1: Supplementary information. (DOCX 448 kb)

Additional file 2: Metadata of downloaded 2094 genomes. (TXT 157 kb)

Additional file 3: MetaCHIP identified 10,255 HGTs from the 2094 genomes. (TXT 805 kb)

Additional file 4: Nucleic acid sequences MetaCHIP identified 10,255 HGTs. (FASTA 16796 kb)

Additional file 5: Taxonomic classification of the human gut and North Sea genome bins. (XLSX 17 kb)

## Abbreviations

AR: Antibiotic resistance
ARDB: Antibiotic Resistance Genes Database
COG: Clusters of Orthologous Groups
GTDB: Genome Taxonomy Database
HGT: Horizontal gene transfer
NCBI: National Center for Biotechnology Information
SCG: Single-copy gene
