# Supplementary material for: MetaCHIP: community-level horizontal gene transfer identification through the combination of best-match and phylogenetic approaches
Source: Microbiome. 2019 Mar 4;7:36. doi: 10.1186/s40168-019-0649-y (PMC6399960; doi:10.1186/s40168-019-0649-y)
Supplement: Supplementary file 1 — Supplementary information. (DOCX 448 kb) [file 40168_2019_649_MOESM1_ESM.docx]

**Supplementary Materials**

**Table S1** Selected 10 *Sphingobium* and 10 *Sphingomonas* genomes

| Class | Strain | abbreviation | BioProject ID |
| --- | --- | --- | --- |
| *Sphingobium* | *Sphingobium baderi* strain DE-13 | SBBA | [300354](https://www.ncbi.nlm.nih.gov/bioproject/PRJNA300354) |
|  | *Sphingobium chlorophenolicum L-1* | SBCH | [50015](https://www.ncbi.nlm.nih.gov/bioproject/PRJNA50015) |
|  | [*Sphingobium cloacae*](https://blast.ncbi.nlm.nih.gov/Blast.cgi#alnHdr_1073516485) JCM 10874 | SBCL | 345589 |
|  | [*Sphingobium herbicidovorans* strain MH](https://blast.ncbi.nlm.nih.gov/Blast.cgi#alnHdr_1173762642) | SBHE | 380772 |
|  | [*Sphingobium hydrophobicum* strain C1](https://blast.ncbi.nlm.nih.gov/Blast.cgi#alnHdr_1240580554) | SBHY | 397800 |
|  | [*Sphingobium indicum* B90A](https://blast.ncbi.nlm.nih.gov/Blast.cgi#alnHdr_1114385888) | SBIN | 50313 |
|  | [*Sphingobium japonicum* UT26S](https://blast.ncbi.nlm.nih.gov/Blast.cgi#alnHdr_292673316) | SBJA | 19949 |
|  | [*Sphingobium* sp. RAC03](https://blast.ncbi.nlm.nih.gov/Blast.cgi#alnHdr_1059502469) | SBRA | 319832 |
|  | [*Sphingobium yanoikuyae* strain SHJ](https://blast.ncbi.nlm.nih.gov/Blast.cgi#alnHdr_1272211495) | SBYA | 239177 |
|  | [*Sphingobium* sp. YG1](https://blast.ncbi.nlm.nih.gov/Blast.cgi#alnHdr_1352285399) | SBYG | 436102 |
| *Sphingomonas* | [*Sphingomonas sp. Cra20*](https://blast.ncbi.nlm.nih.gov/Blast.cgi#alnHdr_1280029430) | SMCR | 418884 |
|  | *Sphingomonas sp. FARSPH* | SMFA | 474545 |
|  | *Sphingomonas hengshuiensis strain WHSC-8* | SMHE | 274606 |
|  | *Sphingomonas koreensis strain ABOJV* | SMKO | 445389 |
|  | *Sphingomonas sp. LM7* | SMLM | 363095 |
|  | *Sphingomonas melonis TY* | SMME | 345324 |
|  | [*Sphingomonas panacis strain DCY99*](https://blast.ncbi.nlm.nih.gov/Blast.cgi#alnHdr_1061224965) | SMPA | 308882 |
|  | [*Sphingomonas sanxanigenens* NX02](https://blast.ncbi.nlm.nih.gov/Blast.cgi#alnHdr_569540043) | SMSA | 213647 |
|  | [*Sphingomonas taxi* strain ATCC 55669](https://blast.ncbi.nlm.nih.gov/Blast.cgi#alnHdr_695169020) | SMTA | 261987 |
|  | [*Sphingomonas wittichii* RW1](https://blast.ncbi.nlm.nih.gov/Blast.cgi#alnHdr_148498119) | SMWI | 17343 |

**Table S2** Selected 10 alphaproteobacterial and 10 betaproteobacterial genomes

| Class | Strain | abbreviation | BioProject ID |
| --- | --- | --- | --- |
| *Alphaproteobacteria* | *Acidiphilium multivorum* AIU301 | AAM | 60101 |
|  | *Ketogulonigenium vulgarum* WSH 001 | AKV | 161161 |
|  | *Mesorhizobium australicum* WSM2073 | AMAU | 47287 |
|  | *Methylocapsa acidiphila* B2 | AMAC | 72841 |
|  | *Methyloferula stellata* AR4 | AMS | 165575 |
|  | *Rhodovibrio salinarum* DSM 9154 | ARS | 84315 |
|  | *Roseobacter litoralis* Och 149 | ARL | 19357 |
|  | *Sphingobium japonicum* UT26S 1 | ASJ | 19949 |
|  | *Starkeya novella* DSM 506 | ASN | 37659 |
|  | *Tistrella mobilis* KA081020 065 | ATM | 76349 |
| *Betaproteobacteria* | *Alicycliphilus denitrificans* K601 | BAD | 50751 |
|  | *Dechlorosoma suillum* PS | BDS | 37693 |
|  | *Gallionella capsiferriformans* ES 2 | BGC | 32827 |
|  | *Herbaspirillum seropedicae* SmR1 | BHS | 47945 |
|  | *Nitrosospira multiformis* ATCC 25196 | BNM | 13912 |
|  | *Ramlibacter tataouinensis* TTB310 | BRT | 16294 |
|  | *Sideroxydans lithotrophicus* ES 1 | BSL | 33161 |
|  | *Snodgrassella alvi* wkB2 | BSA | 167602 |
|  | *Sulfuricella denitrificans* skB26 | BSD | 170011 |
|  | *Tetrathiobacter kashmirensis* WT001 | BTK | 67337 |

**Table S3** Relative abundance of the selected 10 alphaproteobacterial and 10 betaproteobacterial genomes for metagenomic data simulation

| Genome | Relative abundance | | |
| --- | --- | --- | --- |
|  | Replicate 1 | Replicate 2 | Replicate 3 |
| AAM | 226 | 640 | 134 |
| AKV | 669 | 315 | 16 |
| AMAC | 731 | 8 | 261 |
| AMAU | 507 | 292 | 201 |
| AMS | 555 | 332 | 113 |
| ARL | 184 | 554 | 262 |
| ARS | 380 | 290 | 330 |
| ASJ | 399 | 221 | 380 |
| ASN | 246 | 324 | 430 |
| ATM | 338 | 465 | 197 |
| BAD | 468 | 336 | 196 |
| BDS | 651 | 69 | 280 |
| BGC | 456 | 173 | 371 |
| BHS | 652 | 339 | 9 |
| BNM | 938 | 44 | 18 |
| BRT | 97 | 363 | 540 |
| BSA | 478 | 400 | 122 |
| BSD | 332 | 116 | 552 |
| BSL | 497 | 375 | 128 |
| BTK | 345 | 549 | 106 |


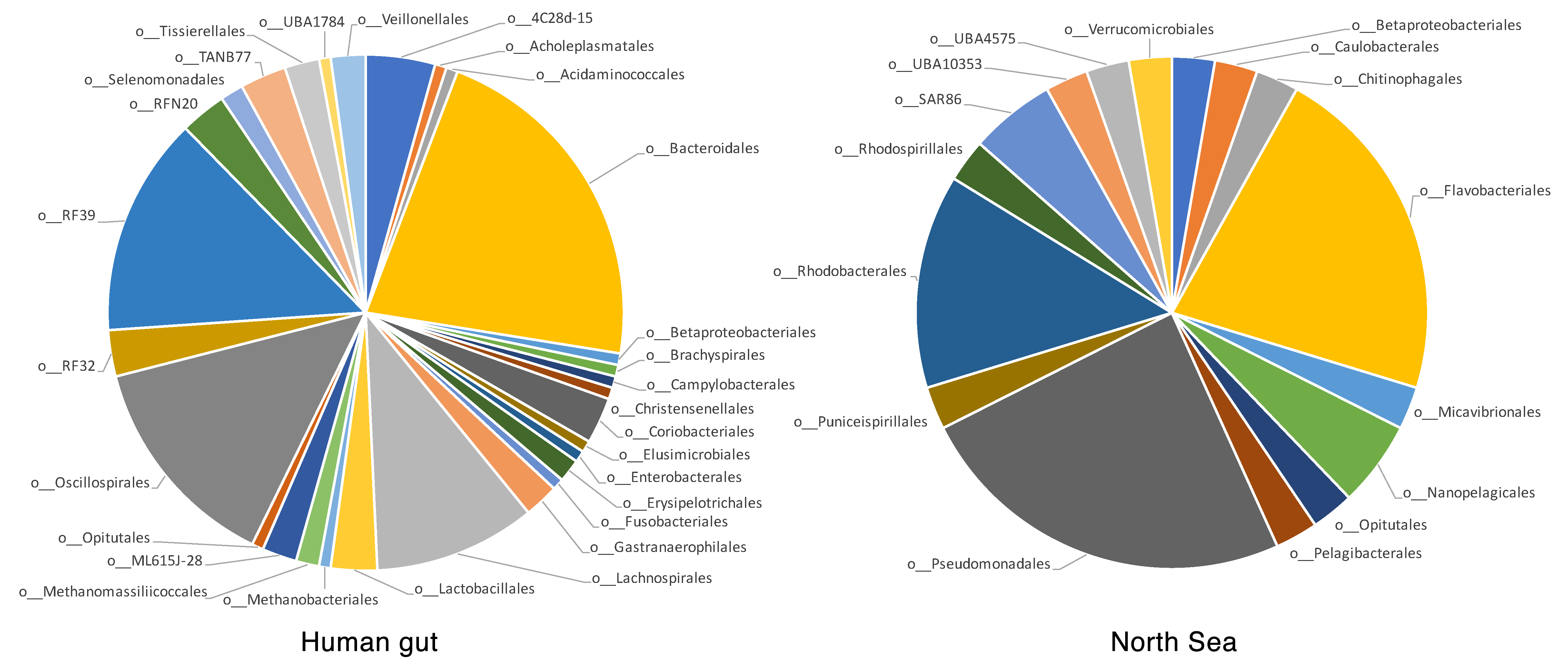


**Figure S1**. Taxonomic classification of the human gut and North Sea genome bins at order level.
